# Analytical Validation of AmpliChip p53 Research Test for Archival Human Ovarian FFPE Sections

**DOI:** 10.1371/journal.pone.0131497

**Published:** 2015-06-30

**Authors:** Matthew J. Marton, Andrew R. McNamara, D. Michele Nikoloff, Aki Nakao, Jonathan Cheng

**Affiliations:** 1 Merck Research Laboratories, Molecular Biomarkers and Diagnostics, Rahway, New Jersey, United States of America; 2 Roche Molecular Systems, Inc., Pleasanton, California, United States of America; 3 Merck Research Laboratories, Clinical Oncology, North Wales, Pennsylvania, United States of America; Virginia Commonwealth University, UNITED STATES

## Abstract

The p53 tumor suppressor gene (*TP53*) is reported to be mutated in nearly half of all tumors and plays a central role in genome integrity. Detection of mutations in p53 can be accomplished by many assays, including the AmpliChip p53 Research Test. The AmpliChip p53 Research Test has been successfully used to determine p53 status in hematologic malignancies and fresh frozen solid tissues but there are few reports of using the assay with formalin fixed, paraffin-embedded (FFPE) tissue. The objective of this study was to describe analytical performance characterization of the AmpliChip p53 Research Test to detect p53 mutations in genomic DNA isolated from archival FFPE human ovarian tumor tissues. Method correlation with sequencing showed 96% mutation-wise agreement and 99% chip-wise agreement. We furthermore observed 100% agreement (113/113) of the most prevalent TP53 mutations. Workflow reproducibility was 96.8% across 8 samples, with 2 operators, 2 reagent lots and 2 instruments. Section-to-section reproducibility was 100% for each sample across a 60 μm region of the FFPE block from ovarian tumors. These data indicate that the AmpliChip p53 Research Test is an accurate and reproducible method for detecting mutations in *TP53* from archival FFPE human ovarian specimens.

## Introduction

The p53 tumor suppressor gene (*TP53*) is one of the most studied genes in cancer, with more than 38,000 articles devoted to its study over the past 25 years [1–3]. The *TP53* gene, which has been dubbed the “guardian of the genome”, is one of the most frequently mutated genes in human solid tumors, including ovarian and lung cancers. The protein product of the *TP53* gene is a transcription factor which is activated in response to a variety of cellular insults including DNA damage [4]. Activated p53 in turn triggers a variety of genetic programs that include cell cycle arrest, DNA repair pathways, and the induction of apoptotic cell death. Functional p53 is critical for controlling genetic stability and the elimination of cells with mutations. Loss of p53 function, as occurs in the Li-Fraumeni syndrome, is associated with a dramatic increase of cancers occurring in various tissues, and at an early age. Although p53 mutations are very common in human cancer, the vast majority are somatic mutations, indicating that they are acquired defects, caused by various types of genotoxic injury.

The International Agency for Research on Cancer (IARC) has amassed a large database (p53.iarc.fr) that catalogs thousands of *TP53* mutations found in a wide variety of human cancers (reviewed in [5]). In the TP53 Database R17 release, approximately 73% of *TP53* mutations in human cancers are single-base missense substitutions that alter a single amino acid in the p53 protein. Many of these mutations stabilize the p53 protein. Approximately 8% are nonsense mutations, 9% are frameshift insertions or deletions, and 4% are silent mutations. Of the unique mutations reported in the IARC database version R17, the 10 most frequent mutations account for about a quarter and most of these mutations reside in exons 5–8, which encode the DNA-binding domains of the protein. Thus strong selection for certain mutations occurs during the oncogenic process.

Because *TP53* is a tumor suppressor gene, any mutation within the gene that results in a loss of function could potentially be tumorigenic (as opposed to oncogene activation at one or a few hotspot regions resulting in gain-of-function related to the oncogenic state). Thus, to determine gene mutation status of a tumor suppressor gene such as *TP53* (*i*.*e*. distinguish between wildtype and mutant), it is necessary to sequence all, or substantially all, of the coding sequence of the gene. Prior to the emergence of massively parallel sequencing (also known as Next Generation Sequencing or NGS), the primary method by which mutations along the entire length of a gene have been identified is by Sanger sequencing, which is laborious for a large gene and lacks sensitivity to detect minor allele frequency mutations. The AmpliChip p53 Research Test represents a convenient and efficient microarray-based method to detect nearly the entire *TP53* sequence [6] and has been deployed in a number of different settings with different sample types. For example, the test has been used with fresh frozen breast tumor tissue in prospective clinical studies to demonstrate prognostic value of *TP53* mutation status [7–8] and that treatment response correlates with TP53 mutation status in early stage breast cancer [9]. One study used the test with fresh frozen ovarian cancer tissue [10]. In addition, it has been used with blood samples from AML patients to show prognostic value [11], and from CLL patients to evaluate the frequency and impact of *TP53* mutations on progression free survival in the Phase 3 REACH trial [12]. There are few reports of use of the assay with FFPE tissue, such as in head and neck cancer tissue [13] and in liposarcoma FFPE tissue [14]. Several groups have compared the performance of AmpliChip p53 Research Test versus Sanger sequencing in non-FFPE tissues [11] [15]. In general these studies concluded that the test had lower failure rate and superior sensitivity compared to Sanger sequencing.

The functional consequence of different *TP53* mutations has been extensively studied. For the relatively uncommon mutations that result in a truncated or absent protein, the phenotype is clearly loss of p53 function. The situation with the more common missense mutations is more complex. In a large majority of these cases, the resultant mutant protein shows dramatic loss of its ability to activate the normal gene targets of p53, indicating a loss of transcriptional activity. In addition, when tested together with wild type (WT) p53 protein, many of the mutant proteins, particularly those with amino acid substitutions in the DNA-binding domains, exhibit a dominant-negative effect on the function of the normal protein. Thus, the marked predominance of missense mutations over nonsense mutations in cancer may reflect the fact that the presence of a mutant protein with a dominant-negative effect on the remaining normal p53 protein is more oncogenic than loss of p53 protein.

In addition to the key role that p53 mutations play in the molecular pathogenesis of cancer, most chemotherapy drugs, hormonal and irradiation therapies depend largely on p53-mediated apoptosis for efficacy [1]. Of note, the majority of human cancers carry abnormalities in the p53 pathway, and hence lack the G1 checkpoint but retain the S- and G2- phase checkpoints [16]. The p53 protein is also believed to mediate the efficacy of small molecule inhibitors of Wee1, a tyrosine kinase involved in regulation of cell cycle checkpoints [17–20], particularly the G2 checkpoint [21]. Cell cycle checkpoints are critical in DNA damage response. The cell cycle is regulated by a series of cyclin dependent kinases (CDKs) which govern checkpoints by causing transient arrest at the G1-, S-, and G2 phases of cycling cells, allowing time to repair the DNA damage or to initiate apoptosis if the DNA damage is too extensive [22]. While DNA checkpoints can protect normal cells from DNA damage, they also reduce the effectiveness of chemotherapy on tumor cells by allowing tumor cells to repair DNA damage induced by the chemotherapy. Thus, selective inhibition of checkpoints in tumor cells is predicted to enhance the efficacy of DNA-damaging agents since mutations will not be repaired.

Wee1 activity can be altered by inhibiting the phosphorylation of its direct substrate (CDC2 Y15 residue). By inhibiting Wee1, small molecular inhibitors of Wee1 potentiate the activity of cytotoxic agents and thus act to sensitize the tumor cell to the cytotoxic agent. p53 deficient cells lacking the G1 checkpoint are predicted to be more dependent on the Wee1-mediated G2 checkpoint. Hence, p53-deficient tumors treated with inhibitors of Wee1 may be particularly susceptible to DNA damage that cannot be repaired due to activity of multiple checkpoints being lost, whereas non-tumor tissue will have normal p53 activity and retain G1 checkpoint activity. Thus, checkpoint abrogation caused by Wee1 inhibition may selectively sensitize p53-deficient cells to anti-cancer agents while sparing normal tissues from toxicity [23]. This potentiation may not take place in all tissue types however, as one study showed the anti-tumor activity of Wee1 inhibition was not dependent p53-dependent in sarcoma [18].

Ovarian cancer represents the leading cause of death from gynecologic malignancies and despite advances in treatment, more than 80% of patients with stage III or IV disease die within 5 years. The majority of patients who present with epithelial ovarian cancer respond well to the initial treatment, but will ultimately experience a recurrence of their disease [24]. Recurrent ovarian carcinoma is divided into two subsets of patients based on their response to initial platinum-based chemotherapy: those with platinum-sensitive disease and those with platinum-resistant disease. Patients with disease free interval longer than 6 months are treated with platinum based combination chemotherapy. For patients with platinum resistant disease chemotherapy has a palliative role [24]. Several non-platinum agents have been tested and demonstrated activity in the treatment of recurrent tumor; however, response rates have only been in the 10% to 30% range, with modest duration [25]. The discovery and development of anticancer therapies with novel mechanisms of action is needed for patients with advanced ovarian cancer resistant to platinum based chemotherapy.

A necessary step in achieving the goal of better treatment outcomes by determining which cancer patients are likely to respond to innovative therapeutic candidates is the development of an assay that can reliably detect p53 mutations. We sought to assess the feasibility of determining p53 mutation status from formalin-fixed paraffin-embedded (FFPE) ovarian tumor specimens using the AmpliChip p53 Research Test. This report describes analytical performance characterization of the AmpliChip p53 Research Test to detect p53 mutations in genomic DNA isolated from archival FFPE human ovarian tumor tissues, specifically: method correlation with sequencing, whole workflow reproducibility, and section-to-section reproducibility. These data demonstrate the ability of the AmpliChip p53 Research Test to detect p53 mutations in ovarian tumors preserved in FFPE.

## Materials and Methods

### Samples, Cell Lines and Ethics Statements

Two cohorts of samples were used in these studies: Cohort 1 consisted of 51 commercially-obtained specimens supplied by Roche Molecular Systems, Inc. Cohort 2 consisted of 65 specimens obtained from the Moffitt Cancer Center supplied by Merck. All specimens were obtained in accordance with applicable regulatory requirements. All patients provided written informed consent. The study was approved by the H. Lee Moffitt Cancer Center and Research Institute Institutional Review Board. Cell lines were obtained from ATCC. S1 Table provides details of Cohort samples and cell lines used in this study.

### The AmpliChip p53 Research Test

The AmpliChip p53 Research Test is a re-sequencing microarray designed to detect single base pair substitutions and single base pair deletions in exons 2 through 11 and their splice sites (2 base pairs before and after each exon) in the *TP53* gene (GenBank X54156). The test includes reagents for sample preparation, PCR amplification and post-PCR hybridization, manufactured by Roche Molecular Systems, Inc., and AmpliChip p53 microarrays manufactured by Affymetrix. The procedure for running the assay has previously been described elsewhere [10] but briefly, one 20 μm FFPE section was scraped from the slide, lysed and DNA amplified in two multiplex PCR mixes, covering all p53 exons. DNA was not quantified prior to PCR amplification because spectrophotometric or fluorescent-derived quantification is not representative of amplifiability. A Sample Integrity Test (SIT) is performed, confirming the presence of a specific amplicon band by gel electrophoresis. Absence of the band is an indication of poor quality DNA and a high likelihood of subsequent chip failure. Amplicons are then combined, fragmented, labeled, and hybridized to the chip surface before scanning. 50 ng of a p53 wild type reference sample (DNA from LoVo cell line; ATCC CCL-229) is analyzed in parallel, and used for background subtraction. A data analysis algorithm examines the chip for quality control probes, and if the data are acceptable, probes for the detection of p53 mutations are examined and mutation calls made.

#### DNA Sequencing

Sanger sequencing was performed by Polymorphic DNA (Alameda, CA). GS FLX sequencing was performed at Roche Molecular Systems, Inc. Briefly, indexed primers, specific for exons 2–11 of *TP5*3, were used for amplification of 10 μL sample lysate or 50 ng of LoVo reference DNA, followed by gel electrophoresis, comparing amplified product to a known mass ladder. Indexed PCR products were purified using Agencourt AMPure SPRI beads (Beckman Coulter) and quantified by Quant-IT PicoGreen (Thermo Fisher) and normalized for loading 200,000 copies/μL of each amplicon per pool of amplicons for 454 sequencing on the GS FLX system as per the GS FLX Sequencing Method Manual (Roche).

For GS FLX data to be considered acceptable, greater than 100 reads per amplicon were required bidirectionally, and a mutation needed to be present at ≥10% to be a valid call.

#### Method Comparison study

Genomic DNA was extracted from one 20 μm FFPE section. Three samples were excluded from Cohort 2 due to borderline malignant potential and two were excluded due to less than 50% tumor content based on pathology review. AmpliChip p53 Research Test results were compared first with Sanger sequencing. Exons with discrepant mutation calls between the AmpliChip p53 Research Test and Sanger sequencing were further characterized by GS FLX sequencing.

#### Metrics for “Positive Agreement” and “Negative Agreement”

The concepts of “positive agreement” and “negative agreement” were used to evaluate agreement with sequencing methods, as defined below. Calculations were performed at an analytical level based on specific mutations calls for the samples. Positive and negative agreement were calculated by comparing AmpliChip p53 Research Test results to Sanger sequencing, followed by GS FLX sequencing to resolve discordant calls between Sanger and the AmpliChip p53 Research Test (Reference Method). The following calculations were used:

1. Failure rate is the number of samples with at least one exon failure divided by the total number of samples tested. For Sanger sequencing, failure was due to either no read or success in only one direction.
2. Mutation-wise positive agreement is defined by
  $${\text{N}}_{\text{Called}}/{\text{N}}_{\text{MutByRefMethods}}$$
  where N_MutByRefMethods_ is the number of mutations identified by at least two methods described in the previous section and N_Called_ is the number of mutations called by chip among those confirmed mutations (N_MutByRefMethods_).
3. Chip-wise negative agreement is defined by
  $${\text{N}}_{\text{n}}/{\text{N}}_{\text{ChipsTested}},$$
  where N_ChipsTested_ is the number of chips tested. N_n_ is the number of chips where all wild type sites confirmed by the reference method are confirmed by chip. If there is at least one mutation called by the chip which was not confirmed by the either Sanger or GS FLX among the 1240 interrogating positions, the chip is considered “not concordant” and not counted towards N_n_. Failed exons and failed chips were excluded from the calculations. Samples with <50% tumor percent were excluded from the Method Correlation Study. Acceptance criteria were established as < 5% sample failure rate after the SIT for FFPE resected tumors; >85% mutation-wise positive agreement; >95% chip-wise negative agreement.

#### Prevalent Mutation Study

To test performance at the most prevalent sites of mutation in *TP53*, 16 previously characterized FFPE tumor blocks (ovarian, breast, lung and parotid cancer blocks with ≥ 50% tumor content) or 22 cell lines known to harbor these particular mutations in *TP53* were analyzed with the AmpliChip p53 Research Test analysis. Slides (a single 5 μm section) or cell line DNA at the concentration of the AmpliChip p53 Research Test’s Reference Standard were processed and tested on the AmpliChip p53 Research Test microarray with 3 replicates per sample. All clinical specimens contained ≥ 50% tumor content and p53 mutations were confirmed by sequencing. Detection rate was defined as the number of clinical or synthetic samples with correct calls divided by the total number of samples tested multiplied by 100.

#### Section-to-Section Reproducibility

Four previously characterized mutants and one wild type specimen from 3 vendors were processed by the AmpliChip p53 Research Test using a single 5 μm section. Genomic DNA was extracted from 12 adjacent sections for each ovarian cancer specimen. Percent tumor content was determined by H&E staining by an independent pathologist for sections 1 and 14 to confirm ≥ 50% tumor content. Mutation calls were compared across section replicates for each sample tested. A total of 60 sections (12 sections x 5 samples) were analyzed for reproducibility:

%sample reproducibility=(number of sections in a sample with the same call divided by number of sections tested per sample)multiplied by100.

#### Whole Workflow Reproducibility Study

Genomic DNA was extracted from 8 consecutive 5 μm sections from 8 randomly selected clinical samples previously characterized by sequencing with ≥ 50% tumor content. Seven samples harbored p53 mutations and one sample had a WT genotype. Two AmpliChip p53 Research Test kit lots were tested by two operators. Each operator processed pre-assigned sections using both sets of kit reagents and two instruments. p53 wild type Reference DNA was included in each run. Reproducibility was evaluated at the level of specific mutation calls and QC metrics. Mutation calls were compared across replicates for each sample tested with each reagent lot, operator and instrument. A total of 64 replicates (8 sections x 8 samples) were analyzed. Percent reproducibility per sample (RP_Sample_) is calculated as follows.

$${\text{RP}}_{\text{Sample}}=\left({\text{N}}_{\text{SameCall}}/{\text{N}}_{\text{Tested}}\right)\;\text{x}\;100,$$

where N_SameCall_ is the number of sections with the same call within a sample, and N_Tested_ is the number of sections tested per sample.

## Results and Discussion

### 1. Method Correlation Study for Ovarian Cancer Specimens

The purpose of this experiment was to assess analytical performance of the AmpliChip p53 Research Test compared to Sanger sequencing on FFPE ovarian cancer samples, using GS FLX sequencing to resolve discrepant results between Sanger sequencing and the AmpliChip p53 Research Test. Although Sanger sequencing is a gold standard for genotyping, it is not ideal for detecting somatic mutations that can be present in low abundance, particularly from the shorter, degraded genomic DNA fragments that are typically obtained from FFPE tissue. An allele-specific PCR approach to query for specific p53 mutations is not practical for all 1240 positions interrogated by the AmpliChip p53 Research Test. The GS FLX sequencing uses emulsion PCR-based clonal amplification and can generate thousands of reads per sample, thus allowing for detection of mutations in low abundance [26]. This methodology is well-suited for shorter, degraded DNA fragments.

In the absence of an established gold standard for mutation detection in FFPE tissue samples, the following criteria for the Method Correlation Study were used:

1. If the chip and Sanger agree on the mutation call, the mutation is assumed to be biological truth.
2. If a mutation is detected only by one method (chip or Sanger), the discrepant base position is examined by GS FLX sequencing.
  - If GS FLX generates a valid result that matches either the chip or Sanger result, the matched result is considered the biological truth.
  - If the GS FLX result does not match the chip or Sanger result, the test is repeated.
  - If GS FLX results in a no call, the base position is excluded from the analytical performance calculation.

#### Cohort 1 Analysis for Analytical Performance

Table 1 lists the 51 samples included in Cohort 1, along with the percent tumor content and any exons for which it was not possible to generate data due to a failure to amplify an exon. The failure rate of the AmpliChip p53 Research Test for Cohort 1 samples was low (2%; (1/51)) and lower than that of Sanger (45.1% or 23/51) (Table 1). Table 2 lists the specific mutations detected by both the AmpliChip p53 Research Test and Sanger. Table 3 and Table 4 summarize the discrepant resolution by GS FLX for 14 mutations detected only by Sanger or the AmpliChip p53 Research Test, respectively.

**Table 1:** Cohort 1 samples and exon failures for the AmpliChip p53 Research Test and Sanger sequencing.

	Failed Exons		
Sample Name	Sanger	AmpliChip	Tumor %
A730	2	0	55
BB031	0	0	85
BB032	2,5	0	85
BB033	2	0	90
BB034	0	0	70
BB035	2	0	90
BB036	3	0	70
BB037	7	0	100
BB038	0	0	70
BB039	0	0	70
BB040	0	0	75
BB041	2	0	75
BB042	7	0	75
BB043	3	0	90
BB044	3	0	90
BB045	0	0	95
BB046	2,4	0	85
BB047	6	4b	90
BB048	2,3,4	0	100
BB049	0	0	85
BB050	0	0	80
C132	2,3	0	55
C237	3	0	75
C241	2	0	50
C243	0	0	65
C256	4	0	90
C268	4	0	70
NE051	0	0	90
NE052	0	0	90
NE053	0	0	80
NE055	0	0	80
NE056	0	0	80
NE057	0	0	80
NE058	2	0	80
NE059	0	0	80
NE060	0	0	80
NE061	0	0	80
NE062	0	0	70
NE063	0	0	70
NE064	0	0	70
NE065	0	0	70
NE066	0	0	70
NE067	6	0	70
NE068	0	0	70
NE069	8	0	60
NE070	0	0	60
NE071	0	0	60
NE072	2	0	60
NE073	3	0	60
NE074	0	0	50
NE075	0	0	50

**Table 2:** The 31 mutations called by Sanger and the AmpliChip p53 Research Test (Cohort 1). Nt Num is the nucleotide position number.

**Sample Name**	**Exon**	**Nt Num**	**Call**
BB033	7	14049	C>T
BB034	8	14487	G>A
BB035	8	14585	C>T
BB036	5	13215	A>T
BB038	6	13338	A>G
BB040	8	14513	C>T
BB041	5	13215	A>G
BB043	6	13413	delT
BB044	6	13346	C>T
BB049	9	14679	A>G
BB050	8	14513	C>T
C237	11	18621	C>T
C243	5	13203	G>A
C268	6	13397	C>T
NE052	8	14585	C>T
NE053	7	14058	G>T
NE055	8	14585	C>T
NE056	4	12139	G>A
NE057	4	12139	G>A
NE058	4	12139	G>A
NE059	4	12139	G>A
NE062	7	14058	G>T
NE063	7	14070	G>A
NE064	8	14585	C>T
NE065	7	14070	G>A
NE066	10	17602	C>T
NE068	7	14070	G>A
NE070	10	17602	C>T
NE072	6	13419	A>G
NE074	7	14069	C>T
NE075	7	14069	C>T

**Table 3:** Discrepant resolution of the 10 mutations called only by Sanger (Cohort 1)

Sample Name	Exon	Nt Num	Sanger Call	Tumor (%)	AmpliChip	454
BB033	4	12148	C>T	90	wt	wt
BB033	4	12263	C>T	90	wt	wt
BB033	4	12279	G>A	90	wt	wt
BB045	8	14498	delT	95	wt	delT
BB048	5	13118	G>A	100	wt	wt
BB048	10	17623	G>A	100	wt	wt
BB048	11	18645	C>T	100	wt	no call
C237	4	12057	C>T	75	wt	wt
C237	4	12153	C>T	75	wt	wt
C243	4	12030	C>T	65	wt	wt

**Table 4:** Discrepant resolution of the 4 mutations called only by the AmpliChip p53 Research Test (Cohort 1)

Sample Name	Exon	Nt Num	Chip Call	Tumor (%)	Sanger	454
C132	6	13346	C>T	55	wt	C>T
BB037	7	14030	A>C	100	fail	wt
BB042	7	14077	delC	75	fail	delC
NE069	8	14585	C>T	60	fail	C>T

31 mutations were called by both methods (Table 2). Among the 10 discrepant base positions that were called WT by the AmpliChip p53 Research Test and p53 mutant by Sanger sequencing, eight mutations were reported as WT based on GS FLX discrepant resolution (Table 3). The AmpliChip p53 Research Test was unable to detect a delT at position 14498 (BB045). One sample remained unconfirmed due to a no call result by GS FLX sequencing (BB048). Among the 4 discrepant base positions that called p53 mutant by AmpliChip p53 Research Test and WT or failed by Sanger in Table 4, three samples were p53 mutant by GS FLX sequencing. One sample was WT at position 14030 by GS FLX sequencing (BB037). Retesting of BB037, BB045 and NE072 confirmed the original AmpliChip p53 Research Test result. Thirteen discrepant calls in exons 4, 5, 6, 7, 8 and 10 were resolved by GS FLX sequencing. In summary, the mutation-wise positive agreement between the AmpliChip p53 Research Test and the reference methods was 97.1% (34/35) and chip-wise negative agreement was 98% (50/51).

#### Cohort 2 Analysis for Analytical Performance

As summarized in Table 5, the failure rate of the AmpliChip p53 Research Test for 60 Cohort 2 samples was 0% and lower than that of Sanger (20%). Table 6 lists the specific mutations detected by the AmpliChip p53 Research Test and Sanger sequencing and Table 7 summarizes the discrepant resolution by GS FLX of 2 mutations in exons 2 and 8 that were detected by Sanger only. Retesting of discrepant samples resulted in the same AmpliChip p53 Research Test call indicating that no sample mix ups or operator error had occurred. Mutation-wise positive agreement was 97.0% (32/33) and chip-wise negative agreement was 100% (60/60).

**Table 5:** Cohort 2 samples and exon failures for the AmpliChip p53 Research Test and Sanger sequencing.

	Failed Exons		
Sample Name	Sanger	AmpliChip	Tumor%
MK002	5,6	0	95
MK012	5	0	96
MK015	3	0	100
MK022	3	0	97
MK023	6	0	95
MK026	2,3	0	95
MK027	2	0	80
MK029	3	0	98
MK044	4,11	0	N/A
MK049	6	0	90
MK050	5	0	90
MK051	4	0	55
MK057	5	0	80

**Table 6:** The 32 mutations called by both Sanger and the AmpliChip p53 Research Test (Cohort 2).

Sample Name	Exon	Nt Num	Call	Tumor (%)
MK001	5	13167	A>G	100
MK003	7	14052	G>T	100
MK004	6	13407	T>G	90
MK005	5	13203	G>A	100
MK006	8	14513	C>T	100
MK007	5	13203	G>A	100
MK008	10	17602	C>T	97
MK011	8	14487	G>A	100
MK015	8	14502	C>T	100
MK016	6	13338	A>G	90
MK017	5	13203	G>A	98
MK018	6	13343	A>T	95
MK022	6	13341	T>G	97
MK024	7	14028	A>G	92
MK025	7	14061	G>A	97
MK026	8	14487	G>A	95
MK029	8	14454	G>T	98
MK031	7	14049	C>T	90
MK032	6	13397	C>T	90
MK036	8	14487	G>A	80
MK038	5	13080	T>C	100
MK039	6	13419	A>G	75
MK041	6	13338	A>G	85
MK043	6	13397	C>T	100
MK045	8	14486	C>T	100
MK047	6	13344	T>C	100
MK053	7	14070	G>A	95
MK054	6	13397	C>T	97
MK060	8	14486	C>T	92
MK061	5	13160	G>A	90
MK064	8	14489	G>T	90
MK065	5	13053	A>C	85

**Table 7:** Discrepant resolution of mutations called by Sanger only (Cohort 2).

Sample Name	Exon	Nt Num	Sanger Call	Tumor (%)	AmpliChip	454
MK020	8	14507	A>G	85	wt	A>G
MK051	2	11746	C>T	55	wt	wt

Combining the Cohort 1and Cohort 2 analytical performance datasets (n = 111), Table 8 shows the overall performance of the AmpliChip p53 Research Test: mutation-wise positive agreement was 97.1% (66/68) and chip-wise negative agreement was 99.1% (110/111), exceeding the acceptance criteria. The failure rate was 0.9% (1/111).

**Table 8:** Summary of analytical performance (Combined Cohort 1 and 2).

	Cohort 1		Cohort 2		Combined	
	(n = 51)		(n = 60)		(n = 111)	
Mutation-wise positive agreement	97.1%	(34/35)	97.0%	(32/33)	97.1%	(66/68)
Chip-wise negative agreement	98.0%	(50/51)	100.0%	(60/60)	99.1%	(110/111)
Failure rate	2.0%	(1/51)	0.0%	(0/60)	0.9%	(1/111)

### 2. Prevalent Mutation Study for Ovarian Cancer Specimens

Given the vast number of possible mutations across the p53 gene, it was not feasible to assess mutation detection at each of the 1240 sites interrogated by the AmpliChip p53 Research Test. Nonetheless, it was important to demonstrate adequate performance of the most prevalent mutations that have been reported in IARC for ovarian cancer. The purpose of this experiment was to determine the reliability of the AmpliChip p53 Research Test to detect the six most prevalent mutations reported in ovarian cancer.

The six most common p53 mutation in ovarian cancer and their mutation frequencies are shown in Table 9. To assess the analytic performance of the AmpliChip p53 Research Test at these specific nucleotide locations, sixteen clinical samples (FFPE) and 22 cell lines (frozen) know to harbor these TP53 mutations were obtained, and analyzed by the AmpliChip p53 Research Test in triplicate if sufficient sample was available. The number of samples with known mutations that could be obtained is listed in Table 9, and result of triplicate testing is shown in Table 10. Details of cell line mutations are in S2 Table. The overall detection rate was 100% (113/113) for clinical samples and cell lines (Table 10). One chip with 175_2 G>A mutation returned no results due to post labeling control failure and was excluded from the analysis. All clinical samples and cell lines were sequence confirmed.

**Table 9:** Samples obtained and tested in the prevalent mutation study.

Codon	WT Codon	Mutant Codon	Clinical Samples	Cell Lines	IARC% Ovarian Cancer
175	CGC	CAC	2	6	4.99%
220	TAT	TGT	2	1	2.58%
248	CGG	TGG	5	2	2.58%
248	CGG	CAG	5	5	2.36%
273	CGT	TGT	0	2	2.25%
273	CGT	CAT	2	6	4.19%

**Table 10:** Accuracy of detection of the top six prevalent mutations by the AmpliChip p53 Research Test. Samples are those from Table 9, tested in triplicate.

Codon	Base Change	IARC% (Ovary)	Clinical		Cell Line		Total Clinical + Cell line	
175_2	G>A	4.99%	5/5	100%	18/18	100%	23/23	100%
220_2	A>G	2.58%	6/6	100%	3/3	100%	9/9	100%
248_1	C>T	2.58%	15/15	100%	6/6	100%	21/21	100%
248_2	G>A	2.36%	15/15	100%	15/15	100%	30/30	100%
273_1	C>T	2.25%	0	n/a	6/6	100%	6/6	100%
273_2	G>A	4.19%	6/6	100%	18/18	100%	24/24	100%
			47/47	100%	66/66	100%	113/113	100%

### 3. Section-to-Section Reproducibility Study for Ovarian Cancer Specimens

Additional non-clinical studies described here illustrate the performance characteristics of manufactured AmpliChip p53 Research Test kits using FFPE specimens from ovarian tumors. Reproducibility studies were performed with two AmpliChip p53 Research Test pilot lots made under GMP at Roche Molecular Systems’ New Jersey manufacturing facility. The completed studies evaluated section-to-section reproducibility and whole workflow reproducibility.

The purpose of this experiment was to evaluate section-to-section reproducibility and robustness of the AmpliChip p53 Research Test across a FFPE tumor block with a single pilot reagent lot and one instrument system, by one operator. Reproducibility was evaluated at the level of specific mutations as shown in Table 11. Section-to-section reproducibility was 100% for each sample across a 60 micron region of the FFPE block from ovarian tumors. Sixty of sixty sections (100%) yielded the expected p53 calls.

**Table 11:** Summary of section-to-section reproducibility

Sample	# Sections tested	# Sections called correctly	% Reproducibility	Call	Mutant Type	Exon
BB040	12	12	100%	C>T	Missense	Exon 8
C243	12	12	100%	G>A	Missense	Exon 5
NE055	12	12	100%	C>T	Nonsense	Exon 8
NE070	12	12	100%	C>T	Nonsense	Exon 10
NE071	12	12	100%	Wild type	n/a	n/a

### 4. Whole Workflow Reproducibility Study for Ovarian Cancer Specimens

The purpose of this experiment was to evaluate the reproducibility and robustness of the AmpliChip p53 Research Test across data sets generated by two operators with two reagent lots and two instruments. As summarized in Table 12, the average analytical reproducibility was 96.8% (61/63). The delT signals for sample BB043 were relatively weak for all sections and the signal for 2 samples was not strong enough to call as mutant. The IARC prevalence for this particular mutation is 0%. Sample BB043, Section 3 was invalid and failed the Sample Integrity Test, but returned a delT call upon retesting from the sample lysate. Sample BB050, Section 7 had an exon 5 failure, but passed on retesting from sample lysate. QC metrics were comparable between operators, instruments and reagent kit lots.

**Table 12:** Analytical reproducibility of AmpliChip p53 Research Test mutation calls.

Sample	Section 1	Section 2	Section 3	Section 4	Section 5	Section 6	Section 7	Section 8	# Sections tested	# Consistent calls	Section Reproducibility
BB034	G>A	G>A	G>A	G>A	G>A	G>A	G>A	G>A	8	8	100.0%
BB043	delT	Wildtype	Invalid	delT	delT	delT	delT	Wildtype	7	5	71.4%
BB049	A>G	A>G	A>G	A>G	A>G	A>G	A>G	A>G	8	8	100.0%
BB050	C>T	C>T	C>T	C>T	C>T	C>T	C>T	C>T	8	8	100.0%
NE051	Wildtype	Wildtype	Wildtype	Wildtype	Wildtype	Wildtype	Wildtype	Wildtype	8	8	100.0%
NE052	C>T	C>T	C>T	C>T	C>T	C>T	C>T	C>T	8	8	100.0%
NE064	C>T	C>T	C>T	C>T	C>T	C>T	C>T	C>T	8	8	100.0%
NE066	C>T	C>T	C>T	C>T	C>T	C>T	C>T	C>T	8	8	100.00%
							E5 failed		63	61	**96.83%**

## Conclusions

The accurate and reproducible determination of *TP53* mutation status of cancer specimens has become increasingly important to the interpretation of research results. The AmpliChip p53 Research Test has been utilized to identify *TP53* mutations in various settings and in several specimen types, such as blood and fresh frozen tumor, but data on its performance in FFPE tissue is lacking. In this study, we advanced the utility of the AmpliChip p53 Research Test by rigorously examining its accuracy and reproducibility in FFPE ovarian tissue. The results confirm the performance of the AmpliChip p53 Research Test as an effective approach for determining *TP53* mutation status and as tool for the orthogonal validation of mutation results determined by other assays. Furthermore, these results may facilitate research in serous ovarian cancer, in which the *TP53* mutation frequency is reported to be near 90% and for which there are number of interventional clinical studies studying the link between *TP53* mutation status and treatment response.

## Supporting Information

S1 Table Samples used in AmpliChip p53 Research Test Studies. (XLSX)

S2 Table Cell Lines Used in the Prevalence Study. (XLSX)
